# Model of Human Fetal Growth in Hypoplastic Left Heart Syndrome: Reduced Ventricular Growth Due to Decreased Ventricular Filling and Altered Shape

**DOI:** 10.3389/fped.2017.00025

**Published:** 2017-02-22

**Authors:** Sukriti Dewan, Adarsh Krishnamurthy, Devleena Kole, Giulia Conca, Roy Kerckhoffs, Michael D. Puchalski, Jeffrey H. Omens, Heather Sun, Vishal Nigam, Andrew D. McCulloch

**Affiliations:** ^1^Department of Bioengineering, University of California at San Diego, La Jolla, CA, USA; ^2^Department of Mechanical Engineering, Iowa State University, Ames, IA, USA; ^3^Pediatric Cardiology, Primary Children’s Hospital, University of Utah, Salt Lake City, UT, USA; ^4^Department of Medicine, University of California at San Diego, La Jolla, CA, USA; ^5^Pediatric Cardiology, Rady Children’s Hospital, University of California at San Diego, San Diego, CA, USA

**Keywords:** HLHS, growth hormone, biomechanics, sarcomeres, computational model, patient-specific modeling, mechanobiology

## Abstract

**Introduction:**

Hypoplastic left heart syndrome (HLHS) is a congenital condition with an underdeveloped left ventricle (LV) that provides inadequate systemic blood flow postnatally. The development of HLHS is postulated to be due to altered biomechanical stimuli during gestation. Predicting LV size at birth using mid-gestation fetal echocardiography is a clinical challenge critical to prognostic counseling.

**Hypothesis:**

We hypothesized that decreased ventricular filling *in utero* due to mitral stenosis may reduce LV growth in the fetal heart via mechanical growth signaling.

**Methods:**

We developed a novel finite element model of the human fetal heart in which cardiac myocyte growth rates are a function of fiber and cross-fiber strains, which is affected by altered ventricular filling, to simulate alterations in LV growth and remodeling. Model results were tested with echocardiogram measurements from normal and HLHS fetal hearts.

**Results:**

A strain-based fetal growth model with a normal 22-week ventricular filling (1.04 mL) was able to replicate published measurements of changes between mid-gestation to birth of mean LV end-diastolic volume (EDV) (1.1–8.3 mL) and dimensions (long-axis, 18–35 mm; short-axis, 9–18 mm) within 15% root mean squared deviation error. By decreasing volumetric load (−25%) at mid-gestation in the model, which emulates mitral stenosis *in utero*, a 65% reduction in LV EDV and a 46% reduction in LV wall volume were predicted at birth, similar to observations in HLHS patients. In retrospective blinded case studies for HLHS, using mid-gestation echocardiographic data, the model predicted a borderline and severe hypoplastic LV, consistent with the patients’ late-gestation data in both cases. Notably, the model prediction was validated by testing for changes in LV shape in the model against clinical data for each HLHS case study.

**Conclusion:**

Reduced ventricular filling and altered shape may lead to reduced LV growth and a hypoplastic phenotype by reducing myocardial strains that serve as a myocyte growth stimulus. The human fetal growth model presented here may lead to a clinical tool that can help predict LV size and shape at birth based on mid-gestation LV echocardiographic measurements.

## Introduction

Hypoplastic left heart syndrome (HLHS), one of the most severe congenital heart defects, occurs when the left ventricle (LV) is not adequate to provide sufficient blood flow to the systemic circulation (1). Despite recent improvements in clinical management, HLHS patients face substantial morbidity and mortality, with a 1-year transplant-free survival of 64–74% (2–4). The role of biomechanics in normal and pathologic cardiac development *in utero* is an understudied topic. Experimental studies suggest that perturbations in biomechanical stimuli during development can result in HLHS (5–7). The treatment of HLHS can be one of the most expensive neonatal diagnoses, so there is a need for improved quantitative approaches.

Fetal growth occurs *via* hyperplasia (cell proliferation by cell division) and hypertrophy (enlargement of cell size by addition of sarcomeres) and is regulated by developmental stage, growth factors, and hemodynamic load (8–11). During early stages of gestation, when the cardiac structures are still developing, growth is highly regulated by growth factors when hemodynamic load is significantly low. However, after 10–14 weeks of gestation, when the process of cardiac looping is complete and cardiac chambers are fully formed structurally, hemodynamic load is gradually increasing and accelerates hypertrophic cardiac growth significantly (5, 8).

Several studies have shown that cardiac morphogenesis and remodeling adapts in response to changes in biomechanical stress or strain (11–16). Experimental studies in isolated cardiomyocytes have reported sarcomere addition in series or parallel leading to cellular hypertrophy in response to mechanical stretch (13, 17, 18). Altered loading conditions significantly affect gene expression changes at the cellular level via proliferation, mechanotransduction, and hypertrophy signaling pathways resulting in increased mRNA and changes in cardiomyocyte size and shape (8, 19–23). Embryonic sheep, chicken, and zebrafish models with decreased ventricular filling also develop ventricular hypoplasia (6, 7, 14, 24, 25). Partial LV inflow obstruction in the fetal sheep model at mid-gestation resulted in an early form of HLHS within 7 days of the surgical procedure as a 30% decrease in cardiac output and a 70% decrease in LV/right ventricle ratio was reported (6, 13, 16, 26, 27). Additionally, studies have shown that restoring blood flow to the LV can “hemodynamically rescue” the chick model of HLHS (26, 28–34). Importantly, fetuses with narrowing or obstruction of the foramen ovale, mitral valve, or aortic valve frequently develop HLHS (35–37).

Based on these observations, it is likely that decreased biomechanical load associated with impaired ventricular filling can lead to ventricular hypoplasia. Obstruction at the level of the mitral valve (stenosis/atresia) or foramen ovale result in decreased diastolic filling of the LV. This perturbed filling could result in decreased passive stretching of ventricular cardiomyocytes, which would alter the biomechanical-mediated signaling response, thereby affecting cellular and organ level growth (13, 17, 18, 23). Given the limited understanding of the molecular pathogenesis of HLHS and the poor outcomes of current treatments, there is an urgent need to characterize the effects of abnormal ventricular filling and cardiac stretch on embryonic cardiomyocyte growth in the ventricle.

Multiscale computational models of LV growth and remodeling have been used to provide insight into the morphogenetic process of cardiac looping in the embryonic chick heart, cardiac growth in the postnatal rat, and the mechanical mechanisms regulating cardiac remodeling in the adult heart (26, 28, 30–33, 38–41). However, there has been limited use of *in silico* models to study ventricular mechanics and growth in human congenital heart disease. While there have been a limited number of simulations examining the blood flow patterns in congenital heart disease patients (42, 43), there have been no reports of computational models of alterations in LV growth and morphogenesis in human HLHS.

Computational growth modeling of healthy and diseased human fetal hearts requires structural and functional measurements that can accurately elucidate physiological behavior of the heart (28, 31, 44). These data can provide unique information in the fetal heart including the 3D geometry, mechanical parameters, and clinical measures of function. To build an accurate computational model, reliable clinical and experimental measurements are necessary at various fetal developmental stages. To contextualize the findings of disease models and to identify the functional differences from a normally developing heart, it is critical to first understand and characterize the growth behavior and mechanical properties of a normal human fetal heart under varying physiological conditions. Therefore, we developed a single ventricle model of an average-sized human fetal heart to calibrate a normal strain-dependent growth law to serve as a reference model. We sought to understand and quantify the effect of mechanical loads on human fetal ventricular growth using patient-specific computational models of HLHS patients derived from fetal echocardiograms. Specifically, based on the experimental observation that cardiac myocytes hypertrophy in response to stretch as a stimulus (13, 45, 46), we test the hypothesis that reduced ventricular filling observed at end-diastole can predict reduced ventricular growth in HLHS patients with an etiology of inadequate mitral valve patency. Computer-aided diagnostics employing predictive patient-specific models of fetal ventricular growth in HLHS could allow for improved prenatal counseling and potential early selection of candidates for *in utero* interventions.

## Materials and Methods

### Model Development Framework

**Figure 1:**
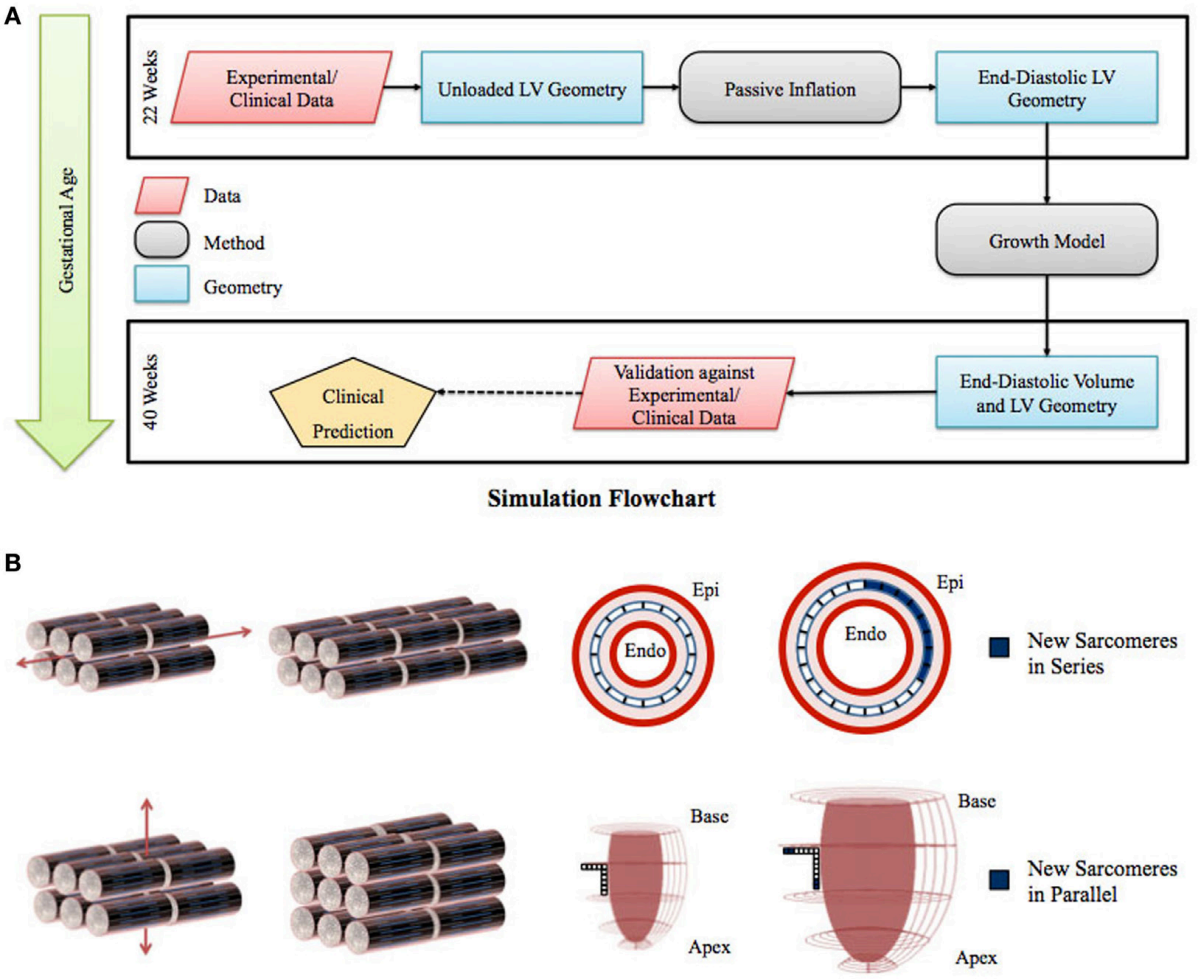
**(A)** Flowchart of left ventricle (LV) fetal growth model. Normative data from fixed fetal hearts were used to construct the unloaded LV geometry at 22 weeks. This geometry was inflated to LV end-diastolic pressure; the passive material properties were adjusted to match the LV dimensions and cavity volume. Growth simulations were then performed to obtain the new LV end-diastolic geometry at each time step. The growth constants were then adjusted to match the LV volumetric growth. The growth in the geometry was validated by comparing the resulting LV end-diastolic dimensions with the dimensions obtained from normal fetuses using echocardiography. **(B)** Graphical representation of the sarcomere addition in series and parallel. Axial strain leads to sarcomere addition in series, which translates to circumferential growth in the LV model. Transverse strain leads to sarcomere addition in parallel, which translates to longitudinal and wall thickness growth in the LV model.

We developed a computational model of human fetal LV growth using the framework of a previously established strain-based growth law (26, 32) (Figure 1). The human LV fetal growth model uses a finite element (FE) model of LV geometry with empirical myocardial material properties adjusted to match the human fetal ventricular measurements at mid-gestation in normal hearts. Experimentally measured LV dimensions were used to generate a FE mesh in a prolate spheroidal coordinate system for describing the ellipsoidal nature of the heart: a thick-walled truncated ellipsoidal shell bounded by inner and outer surfaces. Twenty-four different FE models of idealized LV fetal geometry at 22 weeks of gestation were constructed to optimize the normal fetal LV growth model (Data Sheet S1 in Supplementary Material). The idealized LV geometry was selected based on retrospective error analysis and computation of the least cumulative error for all constraining model parameters and LV shape during growth. The best-fit FE model was used as the reference model for growth simulations.

### Unloaded Fetal LV Geometry

In order to develop the human fetal growth model, we created a FE mesh of the unloaded LV that matches fetal LV dimensions obtained from median dimensions of 14 formalin-fixed human fetal hearts at 22 weeks of gestation (47). Based on the median dimensions, we constructed 24 idealized LV geometries in prolate spheroidal coordinates with 4 radial (endocardium to epicardium) and 5 longitudinal (apex to base) elements. The axisymmetric LV FE mesh used for the simulations consisted of 30 nodes, 20 FEs (5 longitudinal, 4 transmural, and 1 circumferential) with cubic Hermite basis functions for all prolate spheroidal coordinates in the transmural (λ) and longitudinal (μ) directions and linear basis functions for all coordinates in circumferential direction (φ). Cardiomyocyte fiber angles were then incorporated into the model by assigning an inplane angle of −37° (relative to circumferential) at the epicardium, and 83° at the endocardium, with a linear variation across the ventricular wall (48, 49).

### End-Diastolic Fetal LV Geometry

We obtained the end-diastolic fetal LV geometry by passively inflating the unloaded LV mesh. In order to model the resting properties of the myocardium, we make use of the transversely isotropic form of the constitutive model developed by Guccione et al. (50) (Methods S1 in Supplementary Material). The passive material properties of the myocardium were adjusted such that the end-diastolic fetal LV geometry was constrained by clinically measured median values of end-diastolic volume (EDV) (51), EDP (52), and LV dimensions (inner length and diameter) (47, 53) corresponding to 22 weeks of gestation (Table S1 in Supplementary Material).

### Growth Model

The inflated mesh was then set to grow from mid-gestation to birth at a constant EDP with the parameters listed (Methods S2 and Table S2 in Supplementary Material). Briefly, our group previously developed a strain-based volumetric growth model that deforms the stress-free tissue configuration *B*_0_ to a grown configuration *Bg*, which will generally not be stress free (Methods S2 in Supplementary Material) (32). The biomechanical stimuli for growth in these models are derived from maximal strains. The growth deformation gradient tensors are defined with respect to the local fiber orientation (with component *F_ff_* in the fiber direction, component *F_cc_* in cross-fiber direction parallel to the wall, and *F_rr_* the radial component, perpendicular to the two former), which allows for the definition of a transversely isotropic growth tensor (54).

### Geometric Model Optimization

Twenty-four unique FE model geometries were constructed. All 24 FE model geometries have been listed with their parameter values and explicitly shown in Data Sheet S1 in Supplementary Material. These geometries were constrained by the median values of (a) *ex vivo* unloaded fetal LV dimensions of LV length or long-axis (LA), LV diameter or short-axis (SA), and LV wall thickness (WT), as measured by morphometric analysis of 14 fixed hearts (47); (b) end-diastolic LV geometry (LA and SA) as measured by echocardiography (53); and (c) clinical measures of end-diastolic function (EDP and EDV) as measured by *in utero* catheterization (52) and echocardiography, respectively (51), at 22 weeks of gestation (Data Sheet S1 in Supplementary Material). The 24 LV geometries were constructed such that each FE model was unique with various combinations of values of the seven constraining parameters (unloaded LV LA, LV SA, and LV WT dimensions, EDP, EDV, LV LA, and LV SA dimensions at end-diastole, at 22 weeks). Each FE model had to satisfy the condition that every constraining parameter is within the reported measurement/clinical range for that parameter. The idealized LV geometry was selected based on retrospective error analysis of each model (Methods S3 in Supplementary Material). Error analysis was done by computing cumulative *z*-scores for each model, such that each model was fitted to the mean of aforementioned seven constraining model parameters and mean of LV shape growth at incremental time points from mid-gestation to birth. The larger the deviation of the model values from the mean values, the higher the error value for the model. For every model, individual *z*-scores were calculated for all model parameters, i.e., clinical measures of unloaded shape, loaded shape, EDV, and EDP at 22 weeks of gestation, and for LV shape from mid-gestation to term. Individual *z*-scores of all parameters and LV shape growth were summed up for each model to compute the cumulative *z*-score corresponding to the model (see Tables 1 and 2). The model with the lowest cumulative *z*-score was selected as the optimum fetal model.

**Table 1:** ***z*-Scores for geometry and function model parameters at the unloaded and loaded state prior to growth, and cumulative *z*-scores for each dimension during growth from 22 to 40 weeks of gestational stage**.

	Pregrowth (22 weeks)							Postgrowth (22–40 weeks)	
Model	Unloaded dimensions			Loaded dimensions		End diastolic volume	EDP	Dimensions	
				
	Short-axis (SA)	Long-axis (LA)	Wall thickness	SA	LA			SA	LA
1	0.26	0.24	4.08	1.15	1.13	1.62	1.09	5.55	5.88
2	1.77	1.35	3.55	2.46	0.16	0.75	1.09	24.35	10.72
3	0.28	1.30	1.40	1.28	0.04	1.95	1.09	20.24	18.52
4	0.28	1.26	5.05	1.33	0.08	1.94	1.09	8.90	21.87
5	3.28	0.74	1.27	3.77	0.85	1.25	1.09	44.51	12.29
6	4.83	0.64	2.91	5.02	0.99	2.25	1.09	67.86	5.60
7	2.53	0.74	2.04	3.16	0.70	0.40	1.09	32.38	6.80
8	3.24	0.19	1.43	3.71	1.38	1.66	1.09	43.98	9.39
9	1.74	0.19	1.84	2.44	1.22	0.20	1.09	22.56	8.51
10	1.78	0.74	0.10	2.52	0.63	0.46	1.09	21.76	9.81
11	1.46	0.43	2.03	2.21	0.95	0.63	1.09	18.48	5.32
12	2.67	0.37	0.81	3.25	0.63	0.55	1.09	34.72	9.71
13	2.21	0.38	0.21	2.88	1.01	0.11	1.09	27.73	9.56
14	1.93	0.74	0.06	2.65	0.64	0.32	1.09	23.87	9.31
15	2.22	0.74	1.94	2.90	0.68	0.09	1.09	28.16	8.57
16	1.74	0.24	3.76	2.41	1.33	0.02	1.09	23.92	5.24
17	1.18	0.99	0.28	0.53	0.39	0.72	0.74	14.99	9.73
18	0.59	0.79	0.30	1.83	0.67	0.71	0.74	7.11	8.19
19	0.29	0.88	0.39	1.57	0.57	0.80	0.74	12.37	4.21
20	0.29	1.02	0.33	1.58	0.44	1.05	0.74	6.95	8.69
21	0.29	0.96	0.38	1.56	0.49	1.06	0.74	8.41	6.68
22	0.06	0.88	0.36	1.55	0.57	1.03	0.74	9.40	5.11
23	0.00	0.96	0.38	1.32	0.49	0.97	0.74	5.44	6.71
24	0.00	0.84	0.42	1.30	0.62	0.95	0.74	5.29	5.25

**Table 2:** **Cumulative *z*-scores for the model geometries pre- and postgrowth with the minimum cumulative *z*-score highlighted in blue, representing the selected model geometry for reference normal human fetal growth model**.

Model	Cumulative *z*-score		
	Pregrowth	Postgrowth	Total
1	9.57	11.43	21.01
2	11.12	35.07	46.19
3	7.33	38.76	46.09
4	11.03	30.77	41.80
5	12.24	56.81	69.05
6	17.72	73.46	91.18
7	10.65	39.18	49.83
8	12.70	53.37	66.07
9	8.71	31.07	39.78
10	7.32	31.57	38.89
11	8.80	23.80	32.59
12	9.37	44.42	53.79
13	7.88	37.29	45.17
14	7.42	33.17	40.60
15	9.67	36.73	46.40
16	11.47	29.16	40.63
17	4.84	24.72	29.56
18	5.63	15.29	20.93
19	5.24	16.58	21.82
20	5.46	15.63	21.09
21	5.47	15.09	20.56
22	5.19	14.51	19.71
23	4.87	12.16	17.03
24	4.87	10.55	15.41

### Model Simulations

The FE model developed in this study was numerically solved using *Continuity 6.4*, a problem-solving environment for multiscale modeling of cardiac biomechanics and electrophysiology. It is distributed free for academic research by the National Biomedical Computation Resource and can be downloaded at http://www.continuity.ucsd.edu/Continuity.

The different steps in performing the growth simulations are shown in Figure 1. The unloaded fetal LV geometry was inflated to the end-diastolic pressure to obtain the starting LV end-diastolic geometry at 22 weeks of gestation. The growth simulations were then performed by repeatedly applying the growth laws to this end-diastolic geometry to directly compute the grown end-diastolic geometry at each time step. Once the growth simulations were performed, the growth time step that accounts for rate of growth was adjusted to match the normative EDV growth. These growth constants were then kept the same for all subsequent simulations for the different cases.

The non-linear FE models were solved with a modified Newton-Raphson iteration scheme. Integration was performed with 3 × 3 × 3 Gaussian quadrature points. Convergence was reached when both the sum of incremental displacements and the sum of the residuals were lower than 10^−3^ mm and 10^−5^ N, respectively. The Jacobian was calculated and factorized in the first iteration of a new time step and when the solution was diverging. The system of linear equations was solved using SuperLU (55). Boundary conditions in the models were such that the apex was only allowed to move along the LV LA, the base was constrained in longitudinal direction, and the epicardium of the base was constrained in circumferential direction.

Model fits to experimental data were evaluated based on standard error from the data mean or root mean squared deviation (RMSD) from the regression line of the experimental data. The RMSD is calculated using the following formula,

$$\text{RMSD=}\frac{\sqrt{\sum {\left(V_{\text{model}}-V_{\text{regression}}\right)}^{2}}}{n_{\text{data}}}$$

where *V*_model_ is the model-predicted EDV, *V*_regression_ is the EDV calculated using the exponential regression fit to the data, and *n*_data_ is the number of data points.

### Clinical Cases Explored by Model

Once the baseline parameters of the growth model were determined, various growth cases were simulated to determine the effect of volumetric filling, preload, material properties, and shape, on the LV growth. To determine the effect of ventricular filling on growth, the same unloaded geometry was inflated to different preloads by changing the end-diastolic pressure while keeping the material properties constant, and then the growth simulations were performed during which pressures were maintained.

To quantify the effect of ventricular shape and WT on fetal growth, the reference FE model shape was modified by either changing SA to LA ratio or average WT of the LV, prior to inflation. To achieve this, four unloaded geometries were constructed with the same initial volume as the normal unloaded geometry. Two geometries were developed by changing the location of the epicardium nodes uniformly along the LV to yield a thick-walled LV (thick; WT: +30%) and a thin-walled LV (thin; WT: −30%) relative to normal. The other two were developed by manipulating the overall shape of the LV to yield “TallNarrow” (LA:SA: +20%) and “ShortWide” (LA:SA: −20%) geometries. The four geometries were then inflated at a (a) constant preload of 0.75 kPa and (b) constant end-diastolic filling volume relative to unloaded (EDV-*V*_0_) of 383 µL.

### Patient-Specific Simulations

The fetal echocardiograms were conducted in the Pediatric Cardiology Division of Rady Children’s Hospital, San Diego, and Primary Children’s Hospital, Utah, following standard guidelines set by the American Society of Echocardiography. All patient data were retrospectively collected and de-identified. Measurements of the hypoplastic LV were made retrospectively from the recorded echocardiogram clips. Measurements were made in the four-chamber view of the LV internal and external diameters (width) at the base and mid-level, as well as the inner and outer length of the cavity only when the image quality allowed clear definition of the endocardium and epicardium.

Two case studies of HLHS patients were evaluated. The patient-specific FE models were constructed using end-diastolic LV dimensions measured at first clinical time point of study (23.1 and 31 weeks of gestation, respectively). The unloaded geometry was computed using the same material parameter values as the normal fetal heart using the methods described by Krishnamurthy et al. (49). Growth simulations were then performed using the reference growth model parameter values and the LV EDV at second clinical time point of study (30 and 39.1 weeks, respectively) was computed. The predictions from the growth simulations were then independently tested by comparing the simulated LV end-diastolic dimensions at the second clinical time point of study with echocardiographic measurements for the same.

## Results

### Human Fetal LV Reference Model

**Figure 2:**
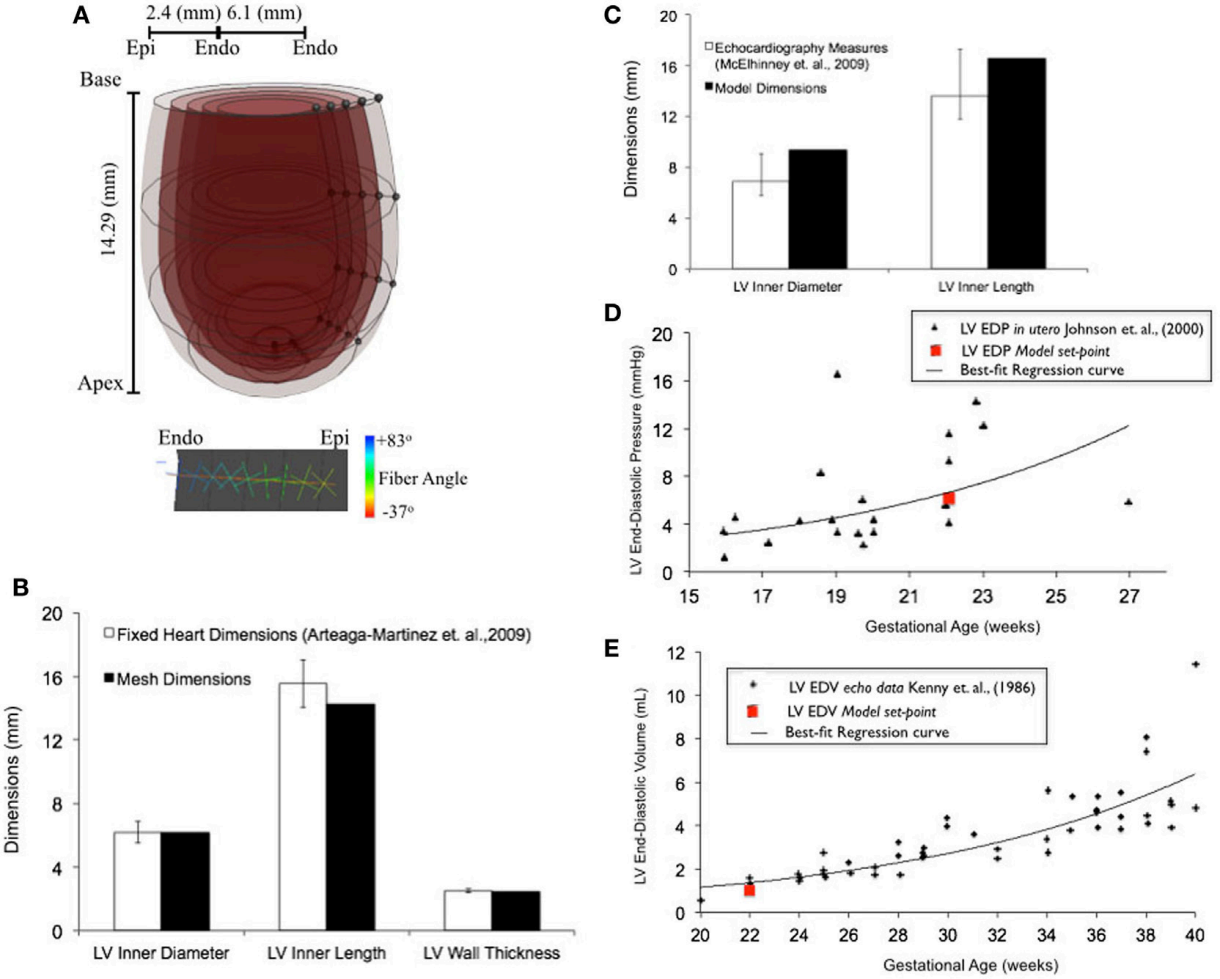
**(A)** Rendered finite element (FE) mesh of unloaded human fetal left ventricle (LV) at 22 weeks of gestational age depicting the four transmural LV wall elements (epicardium to endocardium shade gradations), the five longitudinal elements from apex to base (cross-sectional discs), transmural helical fiber angles from +83° to −37° (endocardium to epicardium; color-mapped sprites) w.r.t. circumferential direction and mesh dimensions for LV inner length, LV inner diameter, and LV wall thickness. **(B)** Bar graph showing comparisons for unloaded LV dimensions between morphometric measurements (white bars) from fixed fetal hearts (47) and FE mesh (black bars) at 22 weeks of gestational age. **(C)** Fitting of simulated LV end-diastolic model geometry (black bars) against echocardiographic measures of LV dimensions (white bars) (53) at 22 weeks of gestational age. Simulation set points (solid red dot) of EDP **(D)** and end-diastolic volume (EDV) **(E)** from human patient data as measured by fetal cardiac catheterization (52) and echocardiography (51), respectively, at 22 weeks of gestational age.

Upon *z*-score analysis of all geometric models constructed (Data Sheet S1 in Supplementary Material), Model 24 resulted with the lowest cumulative *z*-score and was chosen as the working reference human fetal LV model for normal growth (Tables 1 and 2). The geometry of the unloaded FE mesh for the optimum model at mid-gestation (Figure 2A) was within the experimentally reported values of fixed LV hearts at 22 weeks of age (Figure 2B). At end-diastole, the working reference fetal LV model geometry fit at the high-end of the normal clinical range of reported values for LV length and LV diameter (Figure 2C), albeit at the median of clinical values of EDP (5.63 mmHg) and EDV (1.02 mL) (Figures 2D,E).

### Human Fetal LV Growth Model from Mid-Gestation to Birth

**Figure 3:**
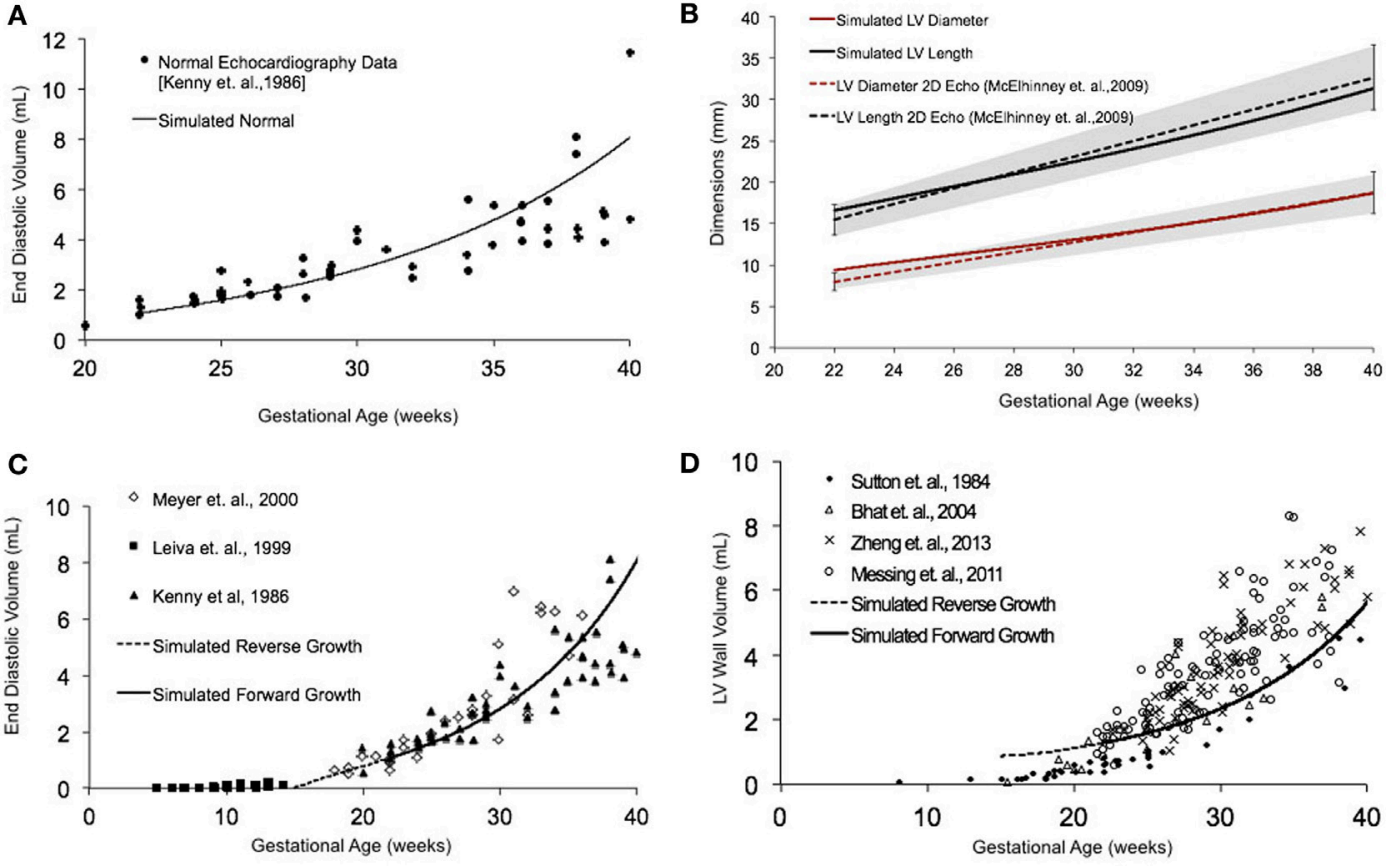
**(A)** Simulated normal human fetal left ventricle (LV) volumetric growth (solid black trace) fitted to normal fetal echocardiographic measures of end-diastolic volume (EDV) (solid black circles) (51) from onset of third trimester of pregnancy (22 weeks of gestational age) to birth (40 weeks of gestational age). **(B)** Validation of simulated LV length (solid black trace) and LV diameter (solid red trace) against clinical echocardiographic measures (dotted lines) (53). Validation of simulated LV EDV **(C)** and LV wall volume **(D)** in forward (solid black line) and reverse (black dotted line) against multiple clinical data sets.

Normal fetal LV growth is quantified from mid-gestation to term and quantified as volumetric and shape growth of the LV cavity and wall. Simulating fetal growth from mid-gestation to birth in the reference human fetal LV model replicated the measured end-diastolic LV cavity volume (Figures 3A,C) and LV geometric dimensions (Figure 3B), and LV wall volume (Figure 3D) to within 15% RMSD error based on echocardiographic measurements (53, 56–61), in both forward and reverse directions, during the third trimester of pregnancy. Specifically, the RMSD between the model-predicted EDV and the exponential regression fit to the EDV data is 0.89 mL. The maximum deviation occurred at 40 weeks, where the percentage of RMSD with respect to the range of data is 13.4%. The RMSD between the model-predicted SA diameter and the linear regression to SA data is 0.606 mm, and the RMSD between the model-predicted LA lengths to the linear regression to the LA data is 1.126 mm. The maximum deviations from data occurred at 22 weeks in both cases and were within 0.55 and 0.61 SDs, respectively. The model was able to accurately predict the physiological unloaded state of the LV at around 12–14 weeks (Figure 3C). However, greater deviation from clinical values is observed for LV wall mass during forward and reverse growth for early-fetal (0–14 weeks) growth (Figure 3D). The RMSD between the model-predicted LV wall mass and the exponential regression to the four different data sets were computed to be 0.99 mL (60), 1.11 mL (57), 1.14 mL (59), and 1.25 mL (58), respectively.

### Human Fetal LV Growth Model Sensitivity to EDV

**Figure 4:**
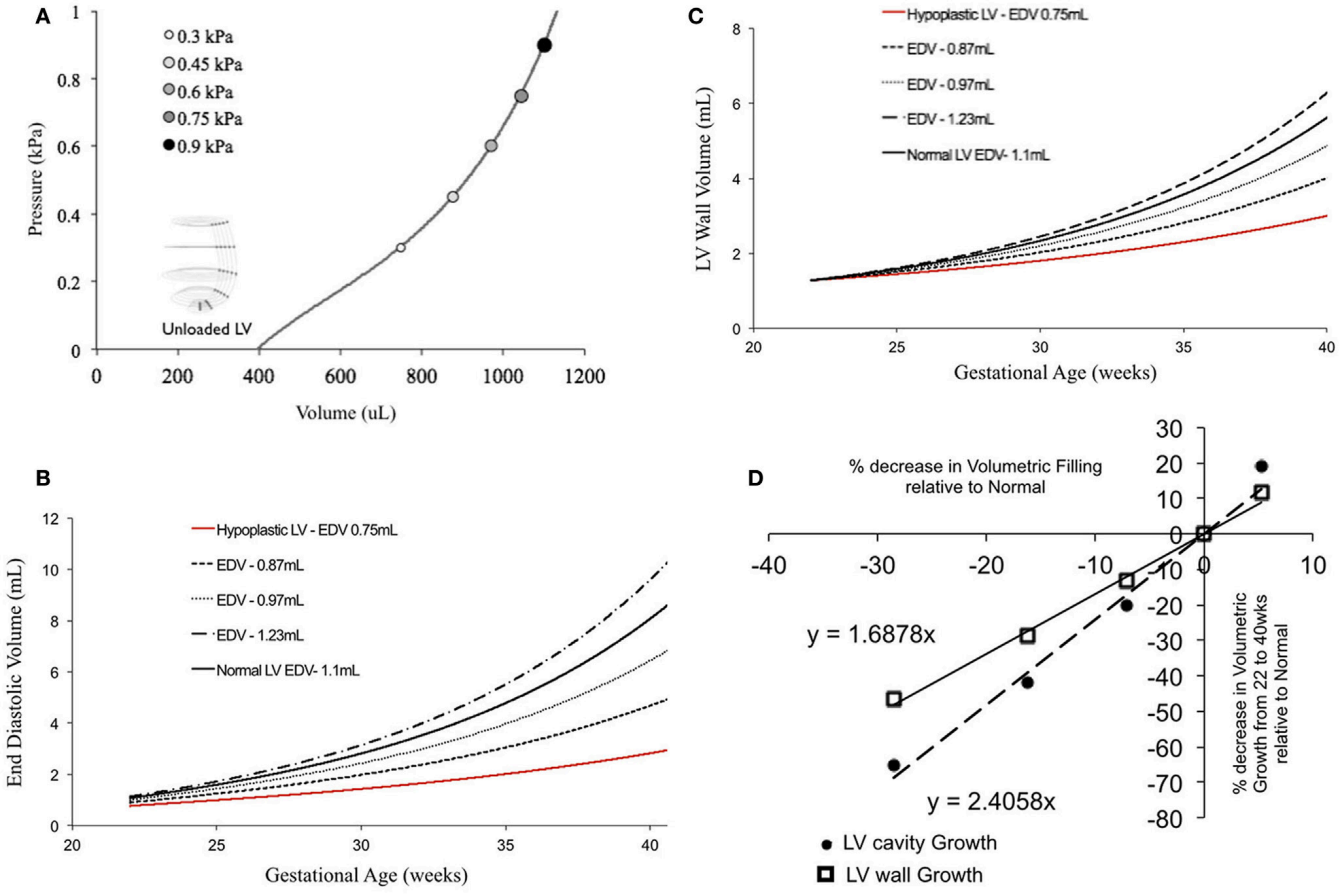
**(A)** Passive inflation of unloaded left ventricle (LV) reference finite element model to varying ventricular filling volumes by varying ventricular loading. Simulated LV cavity volume **(B)** and LV wall volume **(C)** growth under varying ventricular filling conditions. **(D)** Relationship between ventricular filling and LV cavity volume and LV wall volume growth in the reference model.

In fetal echocardiograms, patients with narrowing or obstruction of the foramen ovale, mitral valve, and/or aortic valve frequently develop HLHS. Fetal sheep, chicken, and zebrafish models with decreased ventricular filling also develop ventricular hypoplasia (6, 14, 46). Decreasing the ventricular filling volume in the reference normal growth FE model at 22 weeks, while keeping the material properties of LV constant (Figure 4A), resulted in drastic decreases in LV cavity volume (Figure 4B) and LV wall volume (Figure 4C) during fetal LV growth from mid-gestation to birth. Simulated growth of a hypoplastic LV (reduced ventricular filling) (−25%) resulted in significant reduction in LV EDV (−65%) and LV wall volume (−46%) at birth (Figures 4B,C). A linear correlation was determined between LV filling volume and LV cavity growth/LV wall growth (Figure 4D). Every 10% decrease in LV filling volume at mid-gestation resulted in a 25% decrease in LV cavity volume and 17% decrease in LV wall volume at birth in the reference model.

### Human Fetal LV Growth Model Sensitivity to LV Shape

**Figure 5:**
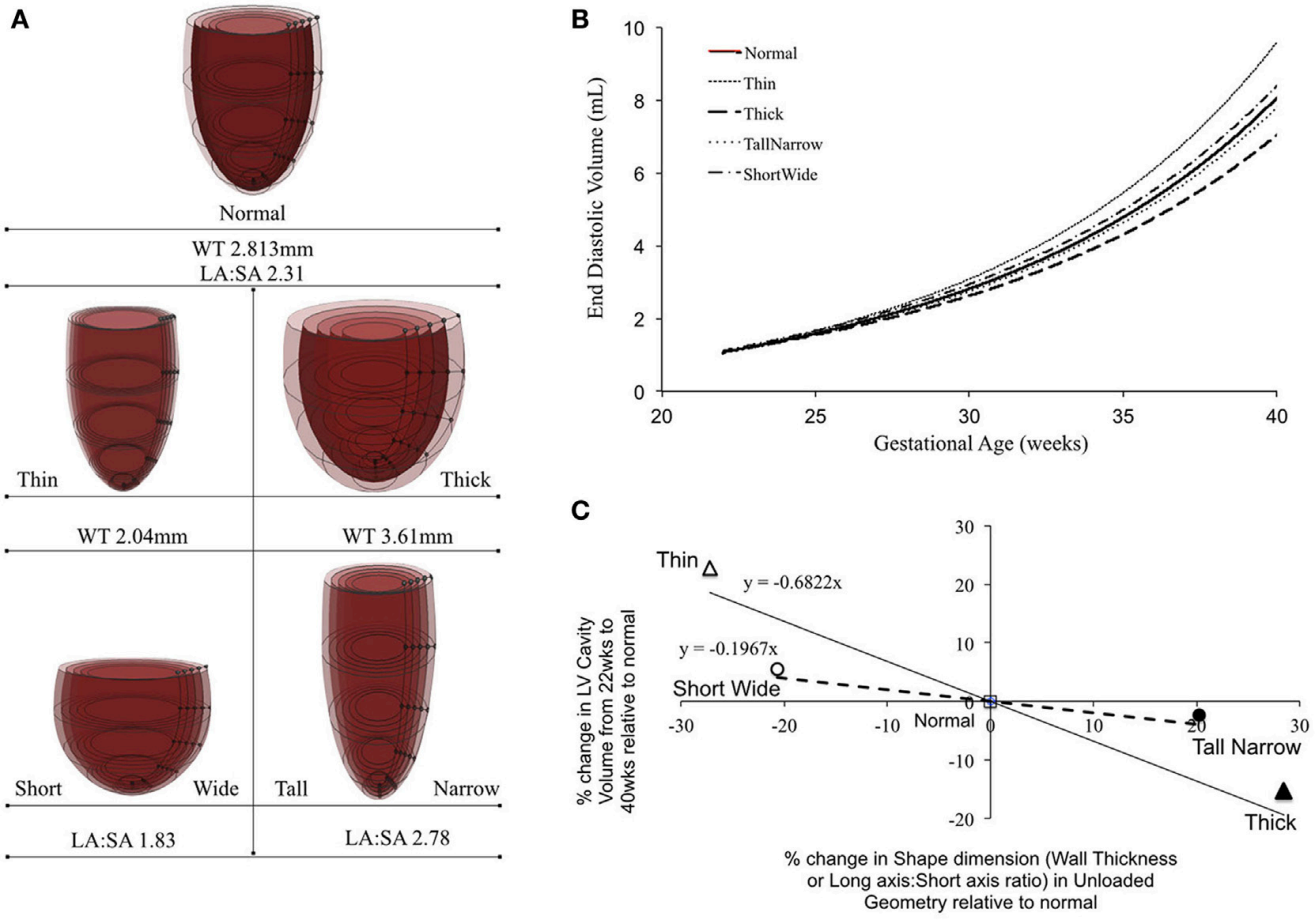
**(A)** Representations of the shape modifications of the reference finite element model by either changing short-axis (SA) to long-axis (LA) ratio or average wall thickness (WT) of the left ventricle (LV), prior to inflation. **(B)** Simulated LV cavity volume growth under varying LV shapes. **(C)** Relationship between LV shape changes and LV cavity volume growth in the reference model.

Patient-specific changes in LV shape, as observed routinely during clinical investigations, can result in deviations from the idealized LV growth and might be prognostic of HLHS phenotype (62). Modifying the reference FE model while maintaining constant initial volume, preload, LV filling volume (EDV-*V*_0_), and material properties (Figure 5A) showed that that thin-walled ventricles grew larger in size and volume than the equivalent thick-walled models (Figure 5B). The effects of changing ventricular length-to-width ratio (Figure 5A) while holding other properties constant were comparatively small. However, the ShortWide LV did grow more than the TallNarrow model (Figure 5B). A steep inverse linear correlation was determined between LV WT and LV cavity growth (Figure 5C). Every 10% increase in LV WT at mid-gestation resulted in a 6.8% decrease in LV cavity volume at birth. Also, a shallow inverse linear correlation was determined between LV LA:SA ratio and LV cavity growth (Figure 5C), wherein, a 10% increase in LA:SA ratio at mid-gestation resulted in a 2% decrease in LV growth at birth.

### Patient-Specific Human Fetal LV Growth Model Case Study 1

**Figure 6:**
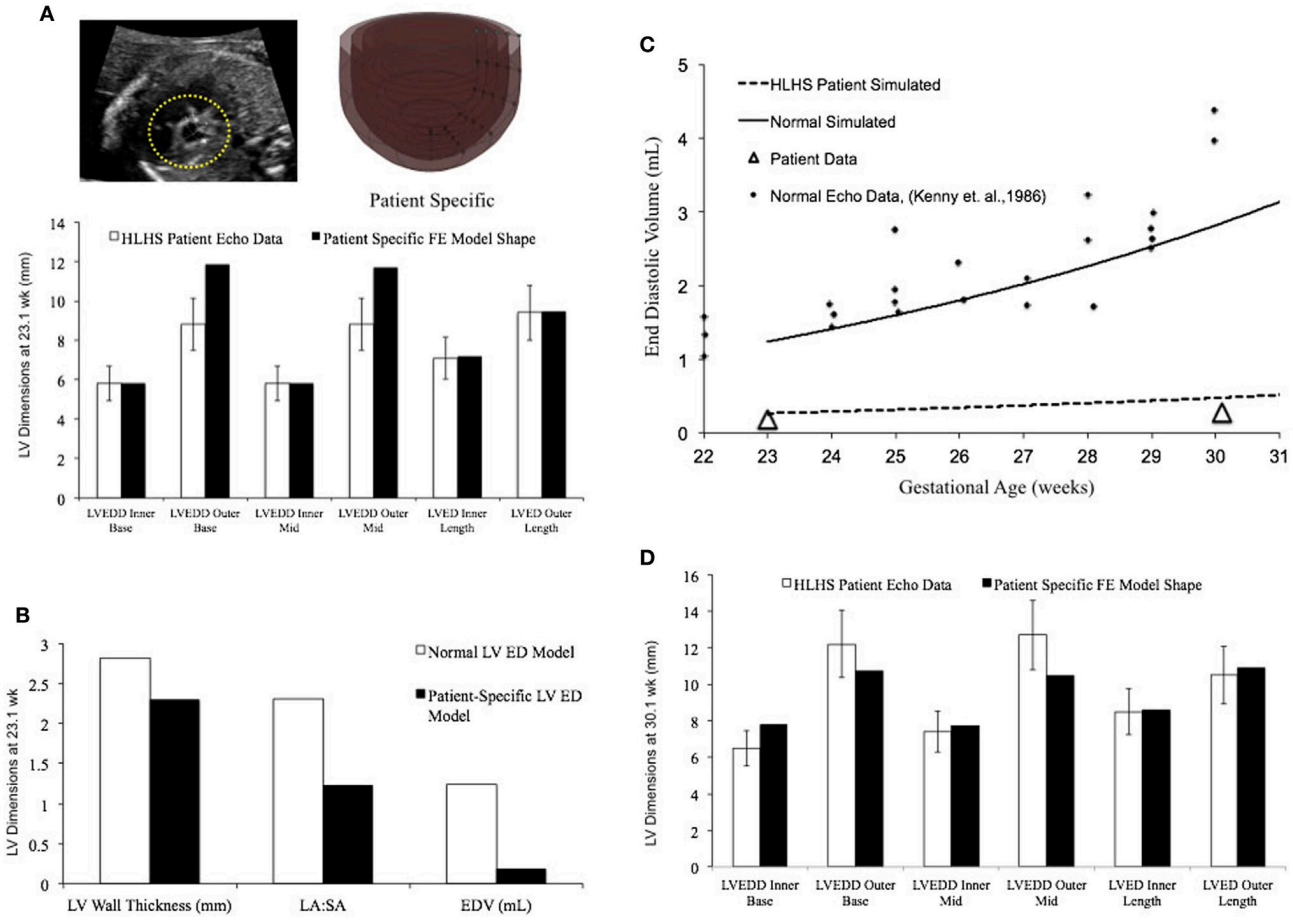
**(A)** Rendering of the patient-specific mesh at end-diastole, generated based on echocardiographic data obtained at 23.1 weeks. Bar graph showing the morphometric match between echocardiographic data (white bars) and patient-specific finite element (FE) model (black bars) at 23.1 weeks. **(B)** Comparison of model shape and ventricular filling between reference FE model of healthy fetal left ventricle (LV) model (white bars) and patient-specific LV model (black bars). **(C)** Simulation of LV cavity growth in patient-specific model (black dotted line) and its comparison to clinically observed growth for the patient (open triangles) and normal LV growth (solid circles and solid line). **(D)** Bar graph showing the morphometric match between echocardiographic data (white bars) and patient-specific FE model (black bars) at 30.1 weeks.

In a blinded case study, using echocardiographic data (for LV geometry) from a severely hypoplastic fetus at 23.1 weeks as the input, we constructed a patient-specific FE model (Figure 6A). The patient-specific FE model was in good agreement with the echocardiography data with highest variability in WT dimension from apex to base (Figure 6A). At end-diastole, the current patient-specific model presented with a significantly lower ventricular filling volume at 0.187 mL, decreased LA:SA ratio at 1.2, and decreased WT relative to a normal healthy LV (Figure 6B). Based on our previous analysis of the reference model, the patient-specific case was predisposed to faster growth as a function of altered geometry (thinner wall and lower LA:SA). In contrast, the patient-specific case was predisposed to much slower growth as a function of lower ventricular filling. The simulated growth of the patient-specific model predicted a severely hypoplastic LV at birth, consistent with the patient diagnosis (Figure 6C). The patient-specific growth model predicted a consistent match in the observed reduction in the measured LV cavity volume (Figure 6C) and end-diastolic LV geometric dimensions (Figure 6D) at 30.1 weeks, to within 15% clinical error based on echocardiographic measurements.

### Patient-Specific Human Fetal LV Growth Model Case Study 2

**Figure 7:**
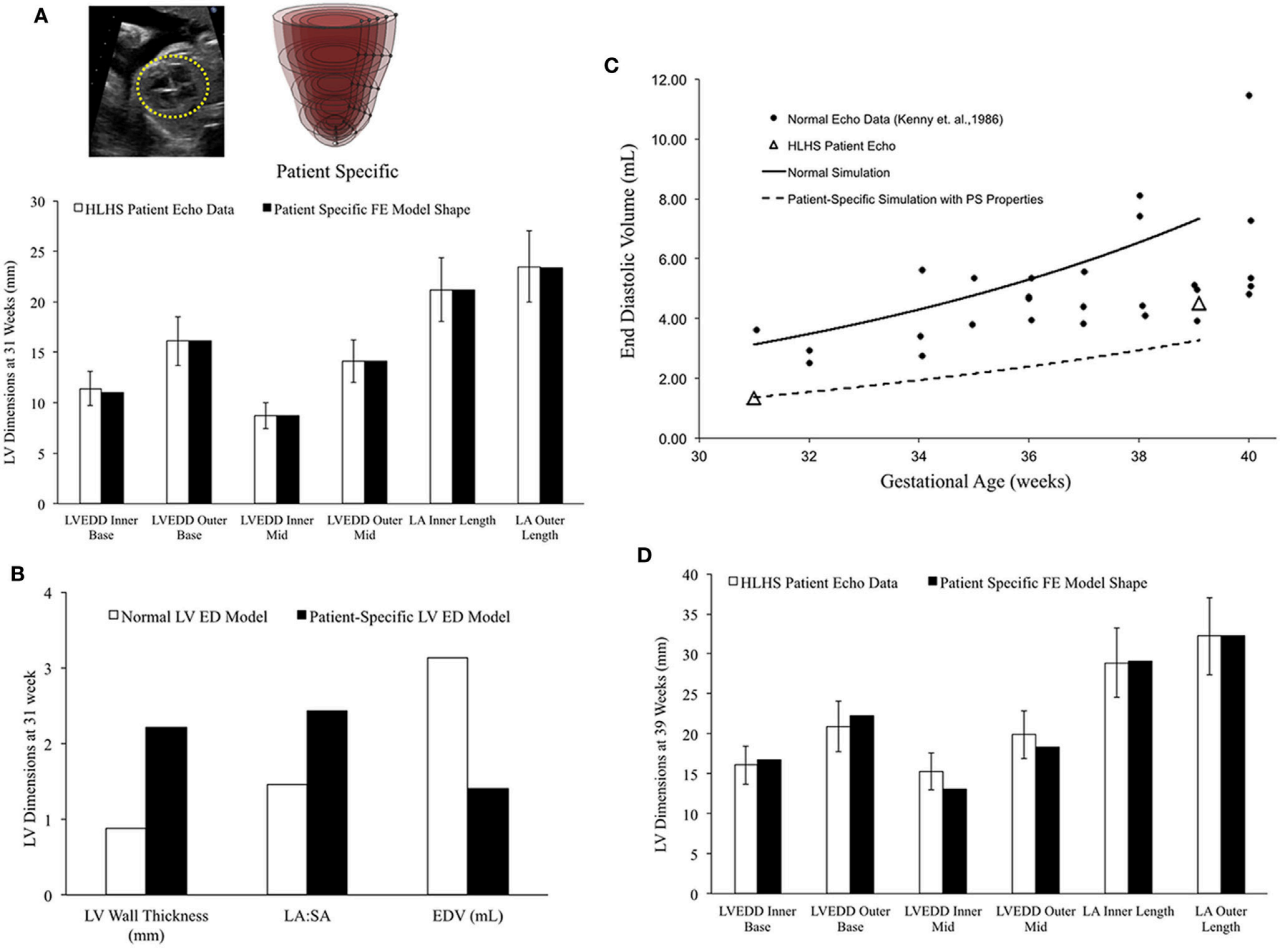
**(A)** Rendering of the patient-specific mesh at end-diastole, generated based on echocardiographic data obtained at 31 weeks. Bar graph showing the morphometric match between echocardiographic data (white bars) and patient-specific finite element (FE) model (black bars) at 31 weeks. **(B)** Comparison of model shape and ventricular filling between reference FE model of healthy fetal left ventricle (LV) model (white bars) and patient-specific LV model (black bars). **(C)** Simulation of LV cavity growth in patient-specific model (black dotted line) and its comparison to clinically observed growth for the patient (open triangles) and normal LV growth (solid circles and solid line). **(D)** Bar graph showing the morphometric match between echocardiographic data (white bars) and patient-specific FE model (black bars) at 39 weeks.

In a blinded case study, using echocardiographic data (LV geometry) from a borderline hypoplastic fetus at 31 weeks as the input, we constructed a well-matched patient-specific FE model (Figure 7A). At end-diastole at 31 weeks, the patient-specific model had a significantly lower ventricular filling volume, increased LA:SA ratio, and increased WT relative to a normal healthy LV (Figure 7B). Based on our previous analysis of the reference model, the patient-specific case was predisposed to slower growth as a function of both altered geometry (thicker wall and higher LA:SA) and lower ventricular filling. The simulated growth of the patient-specific model predicted a borderline hypoplastic LV at birth, consistent with the patient diagnosis (Figure 7C). The patient-specific growth model predicted a consistent match between the measured LV cavity volume (Figure 7C) and end-diastolic LV geometric dimensions (Figure 7D) at 39 weeks, to within 15% clinical error based on echocardiographic measurements.

## Discussion

In this study, we quantitatively investigated, *in silico*, the effect of reduced ventricular filling at end-diastole, in patient-specific models of HLHS, on LV fetal growth. A novel FE growth model of the healthy human fetal LV, using a previously described strain-based growth law, with idealized average geometry at mid-gestation using clinical data, has been presented as a reference human fetal LV growth model from mid-gestation to birth. Model prediction results from the two blinded case studies of HLHS patients are in good agreement with the clinically observed values for LV cavity volume growth and shape changes. To our knowledge, this is the first study to attempt to investigate the biomechanical relationship between LV filling at end-diastole and LV ventricular growth in human fetal hearts with HLHS. The human fetal growth model presented here is a significant step toward the development of a clinical tool that may be used to predict LV size and shape at birth based on mid-gestation LV filling.

### Computational Models of Fetal Growth and HLHS

The specific biomechanical stimuli that trigger cardiac growth are not completely understood. There is general agreement in the field that an increase in stress or strain at organ level and/or cellular level leads to a growth response in the heart (13, 16, 26–28, 30–34). Most cardiac growth models have been formulated, wherein growth is regulated by changes in mechanical stress and/or strain (13, 16, 26, 27). These computational models are based on the experimental observation that at the organ level, volume overload or pressure overload triggers eccentric or concentric hypertrophy, respectively, via changes in regional wall stresses and strains (13, 16, 26–28). Peña et al. constructed simplified ellipsoidal meshes of the human fetal heart from *in vivo* echocardiographic measurements at different gestational ages, which were then used to optimize for the material properties using FE analysis (28). However, they did not directly apply a growth law. They found that while the active tension of the models increased with gestational age, there was not a significant change in the passive material properties. Ohayon et al. used a global stress-based growth law applied directly to the unloaded geometry to simulate global human fetal LV growth (26, 28, 32). Though able to predict the growth of the fetal LV, this model is not based on sarcomere addition along the fiber and cross-fiber direction that happens in cardiac growth. In this model of fetal growth, interpreting the direct effects of stresses and strains along the local fiber directions on LV growth are difficult as global growth has be transformed to the local fiber directions.

The changes in LV WT are mediated by cellular remodeling of cardiomyocytes via sarcomere addition in series or parallel. We applied a previously described strain-dependent growth law acting locally at each gauss point based on the fiber and cross-fiber strains, which has been used to describe both eccentric and concentric remodeling in dogs and neonatal rat growth, in our fetal heart model (31, 51, 52, 56, 61). Earlier studies have reported a monoexponential increase in fetal LV EDV and a linear increase in fetal systolic and diastolic pressures during the course of human gestation (26, 32, 63). Thus, it would suggest that fetal heart growth is largely driven by volume overload as the biomechanical stimuli. Accordingly, growth in our model is driven via maximal fiber and cross-fiber strains at each gauss point. In addition, decrease in ventricular flow is routinely documented in the experimental models and clinical studies of HLHS.

The single ventricle model for normal fetal growth is a significant step toward building subject-specific models based on fetal echocardiography data. To the best of our knowledge, this is the first computational model that describes LV growth behavior in the human fetus by integrating information on LV geometry and function from multiple clinical measurements and predicts patient diagnoses based on mid-gestation echocardiographic geometry. Our fetal human growth model is based on idealized LV geometry at mid-gestation and is able to replicate the later-gestation fetal LV volumetric and geometric growth (size and shape) observed clinically with less than 15% error. Fetal LV dimensions obtained from retrospective echocardiographic images are valuable measurements as they provide routine clinical information about ventricular structure in HLHS patients (49, 51, 52, 56, 61). Fetal ventricles are of smaller scale relative to adult ventricles, which compounds the difficulty of taking accurate measurements from echocardiography, especially for LV WT measurements. This was reflected in the significant variation within the four experimental data sets of fetal LV wall mass (57–60). All four data sets employed different experimental techniques to acquire data that would have led to technical variability within these data. Of the four data sets for WT, our model was able to match the results of only one data set within acceptable values (60). Notably, St John Sutton et al. was the only group to compare their *in vivo* measurements to explanted LV mass measurements (60). More validation with serial data consisting of paired measurements for LV dimensions (LA, SA, and WT) will be required to improve model results given the scatter in data. This becomes pertinent as LV growth is strongly influenced by WT in our model.

### Patient-Specific Human Fetal LV Growth Model Case Studies

In case of subject-specific data obtained from echocardiograms of fetuses diagnosed with HLHS, measurements were only made when the structures were visibly clear and delineated. Nonetheless, there is the possibility of introducing error due to intraobserver variability. Additionally, error can be greater in hypoplastic LV measurements due to their smaller LV size relative to normally developing LV. In order to assess LV geometry accurately for these case studies, six geometric measurements of LV dimensions were provided at different LV planes from apex to base from the 2D echocardiogram four-chamber view. For both patient-specific cases, model predictions matched the clinical data for LV EDV and shape reasonably well. Specifically, the model predicted the shape better for the borderline HLHS case than the severe HLHS case. Interestingly, the biggest discrepancy in shape results for the borderline HLHS case was observed in the LV WT growth. Additionally, the prediction for EDV was much better for the severe HLHS case than for the borderline HLHS case. Nonetheless, in both cases, the model was able to predict the clinical diagnosis of the fetal subject. Ideally, using MRI data and LV inflow, data would allow for more constraints on the developed mesh and, therefore, a more faithful patient-specific geometry. However, early-fetal MRI is a developing field and not a routine clinical procedure as yet. Additionally, LV inflow data were not available for the current case studies. Also, measurements at more than two time points would be valuable in validating the patient-specific model and its predictive capability. In future studies, protocols need to be developed to ensure consistent methods between patients and, if possible, reduce manual error by having multiple experts obtain measurements.

It is noteworthy that the ventricular geometry was imaged at end-diastole when the heart experiences a significant amount of load. An unloading algorithm developed by Krishnamurthy et al. was used to predict the unloaded configuration of the 3D FE model under normal preload and passive material properties, which may not hold true for the patient-specific case (49). This unloaded LV configuration is important computationally and biomechanically, as it serves as the reference unstressed state for calculation of the developed strains in the model. However, the predicted unloaded geometry is able to successfully deform to the measured end-diastolic geometry, demonstrating promising results. Repeating this with a larger set of patients would serve to validate the algorithm as well as the ability of the growth model to predict dimensions at a future time point.

In addition to ventricular filling at EDV, altered shape plays a significant role in altering local strain distribution contributing to LV growth in our model. A greater understanding of the strain distribution experimentally may shed insight into the mechanism underlying the significant wall thickening observed in hypoplastic hearts. Earlier studies report variable systolic strain distribution in hemodynamic chick model of HLHS (28, 49). Further investigation into the role of LV shape and diastolic strain distributions along with myocardial passive material properties is merited to fully comprehend mechanisms underlying HLHS. We quantified the effect of both shape and ventricular filling on LV growth in our model. Once the patient shape is accounted for by using echocardiography data, then based on volumetric filling and shape changes, one can attempt to predict normal and hypoplastic LV growth by using our model. In the future, it would be invaluable to generate a biventricular mesh of the fetal heart with fetal circulation in order to improve the physiological relevance of the model as well as understand the interactive effects between the ventricles in a normal and diseased state. This would be specifically useful in a clinical case such as HLHS where the right ventricle often compensates for the compromised structure and function of the LV.

Model assumptions and limitations:

(a) The simplified ellipsoid shape of the LV used for the reference normal model of fetal growth represents an idealized geometry of the LV. Even in “patient-specific” cases, the FE model is a simplified axis-symmetric representation of the actual LV geometry derived from six-planar measurements of 2-D echocardiography in the four-chamber view. However, the volume calculations and shape calculations based on this LV geometric approximation match the clinical data reasonably well.
(b) The FE model assumes an initial stress-free state of the myocardium and residual stresses are not factored in. More human fetal data are needed to substantiate these assumptions.
(c) In fetal heart growth, cell proliferation substantially contributes to cardiac growth (24, 44, 64). Studies in animal models of sheep have attempted to quantify the contribution of hyperplasia to cardiac growth and shown that this decreases significantly during the third trimester of pregnancy. However, these data and the kinematics of this process, specifically in human fetal hearts, still remain to be elucidated.
(d) We assume that there is no change in passive material properties during growth. An earlier study by Peña et al. supports this (28).
(e) The kinematics of LV growth allows the radial displacement of the base while the apex is free to move.
(f) Growth of the heart is mediated by loading and biomechanical tissue strain, without other stimuli such as growth hormones.

### Clinical Perspective

Hypoplastic left heart syndrome can be diagnosed by fetal echocardiography between 18 and 22 weeks of gestation (44, 64, 65). However, borderline cases of HLHS can go undetected in many early to mid-gestation fetal exams. Studies have reported that neonates with prenatal diagnosis of HLHS show improved hemodynamic stability in addition to providing the opportunity to plan management and counseling for the family (64, 65). Patient-specific computational modeling of developing fetuses with HLHS could serve to improve prenatal diagnosis by providing insight into the biomechanics and growth behavior of the affected ventricle. The methods developed in this study aim to facilitate understanding of fetal growth behavior undergoing normal development and provide a benchmark model for normal growth in the human fetal LV, enabling comparison with patient-specific fetal LV models. Ultimately, with further testing and refinement, the model has potential to aid a clinician in counseling, surgical planning, and management of HLHS with consideration of rescue options for borderline cases of HLHS.

## Conclusion

The human fetal growth model is a novel tool that may be used to understand biomechanical mechanisms underlying HLHS and ultimately quantitatively predict the degree of LV hypoplasia to potentially guide timing of clinical intervention aimed at rescuing the hypoplastic LV in HLHS patients.

## Author Contributions

SD is the lead author who conceptualized the idea, researched the study topic, designed the study, ran the simulations, and wrote the paper. AK, DK, and GC significantly contributed by assisting in study design, running simulations, writing the manuscript, and completing the study. RK formulated the growth law. MP and HS provided the clinical data and assisted in editing the manuscript. JO, VN, and AM supervised the study and provided with all resources to do so.

## Conflict of Interest Statement

AM and JO are co-founders of and have an equity interest in Insilicomed, Inc., a licensee of UCSD software used in this research, and they serve as scientific advisors to Insilicomed, Inc. Some research grants to AM and JO, including those acknowledged here, have been identified for conflict of interest management based on the overall scope of the project and its potential benefit to Insilicomed, Inc. They are required to disclose this relationship in publications acknowledging the grant support; however, the research subject and findings reported here did not involve the company in any way and have no known relationship to the business activities or scientific interests of the company. The terms of this arrangement have been reviewed and approved by the University of California San Diego in accordance with its conflict of interest policies. The other authors have no competing interests to declare.
