# Supplementary material for: Model of Human Fetal Growth in Hypoplastic Left Heart Syndrome: Reduced Ventricular Growth Due to Decreased Ventricular Filling and Altered Shape
Source: Front Pediatr. 2017 Feb 22;5:25. doi: 10.3389/fped.2017.00025 (PMC5319967; doi:10.3389/fped.2017.00025)
Supplement: Supplementary file 1 [file Presentation_1.PDF]

Supplemental Methods

Model of Human Fetal Growth in Hypoplastic Left Heart Syndrome: Reduced Ventricular Growth Due to Decreased Ventricular Filling and Altered Shape

Sukriti Dewan[1], Adarsh Krishnamurthy[1,3], Devleena Kole[1], Giulia Conca[1], Roy Kerckhoffs[1], Michael D. Puchalski[4], Jeffrey Omens[1], Heather Sun[2], Vishal Nigam[2*] ,Andrew McCulloch[1*]

[1]Dept. of Bioengineering, University of California at San Diego, La Jolla

[2]Pediatric Cardiology, Rady Children's Hospital, University of California at San Diego

[3]Dept. of Mechanical Engineering, Iowa State University, Iowa

[4]Pediatric Cardiology, Primary Children's Hospital, University of Utah

Supplemental Methods

S1. End-diastolic Fetal LV Geometry and Passive material properties

In order to model the resting properties of the myocardium, we make use of the transversely-isotropic form of the constitutive model developed by Guccione *et. al.* (1). The equation for the strain energy for this constitutive model is shown in Equation (3). Since there is no reliable information with which to estimate the anisotropy or non-linearity of the myocardial material properties, the exponent parameters, or anisotropy of the original model were not adjusted. To account for the softness of the fetal myocardium, the multiplicative factor c_{pas} was adjusted to match the LV EDV. In Equation(3), E_{ff} corresponds to fiber component of the Lagrangian Green's strain tensor; the subscripts *f,c,r* correspond to the fiber, cross-fiber and the sheet-normal or radial directions, respectively. The stress scaling coefficient c_{pas} determines the stiffness of the material while the exponents control the non-linearity of the stress-strain relationship. The default parameters for the transversely-isotropic constitutive model computed by Guccione *et. al.* (1) resulted in too stiff stress-strain relations for fetal models. Hence, the scaling parameter (c_{pas}) was scaled to fit the end-diastolic pressure volume relations in the fetal model.

$$\mathbf{C} = \mathbf{F}^T\mathbf{F} \tag{1}$$

$$\mathbf{E} = \frac{1}{2}\left(\mathbf{C} - \mathbf{I}\right) \tag{2}$$

$$S = c_{pas} e^{\left(b_f E_{ff}^2 + b_t\left(E_{cc}^2 + E_{rr}^2 + 2E_{cr}^2\right) + 2b_s\left(E_{fc}^2 + E_{fr}^2\right)\right)} \tag{3}$$

An optimization was performed to adjust the c_{pas} to match the fetal filling curve. Following inflation to median fetal LV EDP (2), the resulting LV EDV was compared with the median clinical EDV at 22 weeks gestation (3). If EDV was lower than the clinical measure, then c_{pas} was lowered. The process was iterated until the simulated EDV matched the clinical EDV.

S2. Growth model

The growth model is based on the multiplicative decomposition of the deformation gradient **F** into growth and elastic components (Equation (4)). Based on the method by Rodriguez *et. al.* (4), the growth deformation gradient $\mathbf{F}_g$ corresponds to the deformation between the unloaded geometry, $\mathbf{B}^{\mathbf{0}}$, at the beginning of growth simulations to the unloaded configuration at a growth state, $\mathbf{B}_t^0$, where local kinematic compatibility conditions are not imposed. The deformation gradient $\mathbf{F}_e$ describes the deformation between unloaded geometry $\mathbf{B}_t^0$ and the grown end-diastolic geometry, $\mathbf{B}_t^p$, at end-diastolic pressure p. Cauchy stress $\boldsymbol{\sigma}(\mathbf{F}_e)$ in the tissue depends on this elastic deformation, while $\mathbf{F}_g$ describes a plastic deformation (4-7).

$$\mathbf{F} = \mathbf{F}_e \mathbf{F}_g \tag{4}$$

The biomechanical stimuli for the growth were derived from end-diastolic strains. The deformation gradient tensors were defined with respect to the local fiber orientation (with component F_{ff} in the fiber direction, component F_{cc} in cross-fiber direction parallel to the wall and F_{rr} the radial component, perpendicular to the two former), which allows for the definition of a transversely isotropic (diagonal) growth tensor. The cumulative growth deformation gradient tensor $\mathbf{F}_g^n$ was updated each growth step with the incremental growth deformation gradient tensor $\mathbf{F}_{g,i}$ (5):

$$\mathbf{F}_g^n = \prod_{i=1}^{n} \mathbf{F}_{g,i} \tag{5}$$

$$\begin{aligned} F_{g,i,ff} &= \beta_f E_{ff} \Delta t + 1 \\ F_{g,i,cc} &= \left(\beta_c E_{cc} \Delta t + 1\right)^{1/2} \\ F_{g,i,rr} &= F_{g,cc} \end{aligned} \tag{6}$$

In Equation (6), $F_{g,i,ff}$ describes incremental growth in the fiber direction due to the addition of sarcomeres in series, while for $F_{g,i,cc}$ and $F_{g,i,rr}$ it is assumed that sarcomeres are added equally in parallel in both cross-fiber directions. Because the cellular cross-

sectional area increases due to a thickening stimulus, the square root is taken of the growth stretch ratio in cross-fiber directions such that the volumetric growth remains linearly related to the stimulus. β_f and β_c are growth rate constants in the fiber and cross-fiber direction, respectively and Δt is the time step. The growth equations were setup such that even a small strain will induce growth. This is because the hemodynamic load is low in the fetal heart that would lead to approximately zero average strains with respect to an unloaded reference state. In addition, it was found that isolated postnatal rat myocytes normalize resting sarcomere length; the growth law assumes the same growth mechanism for growth in the human fetal heart. Deviation from cross-fiber strain E_{cc} (parallel to the wall) was chosen as a stimulus for cross-fiber growth – and not radial E_{rr} – because of the experimental finding that contractile protein synthesis is up regulated (8-10) with passive stretch in cross-fiber direction. Optimization of growth constants and time step for human fetal growth was done by calibrating gestational periods and LV cavity growth to human gestational period and experimentally observed LV growth (3), while maintaining the ratio of fiber and cross-fiber growth rates as previously described by Kerckhoffs *et. al.* (11).

S3. FE Model optimization

With the 24 FE models developed, a statistical analysis was conducted in order to determine the best-fit geometry satisfying all of the aforementioned criteria of unloaded LV shape at mid-gestation, EDP, EDV, LV shape at end-diastole at 22weeks and accuracy of prediction of LV shape and size from 22weeks to 40weeks. The previously described experimental and clinical data was used to calculate mean values and standard deviation for normal fetal LV dimensions (short axis; long axis; and, when applicable, an average of wall thickness at the base, mid, and apex level) and end-diastolic volume from mid-gestation to term (2,3,12,13). End-diastolic pressure mean value and standard deviation were calculated at mid-gestation and assumed to remain constant throughout gestation. The same dimensions and measures of function were extracted for each of the 24 developed LV geometries referred henceforth as Models 1-24. For geometries developed prior to Model 19, the inner diameter was obtained from the base level. For

Model 19 and consequent geometries, the inner diameter was extracted from the more clinically relevant level (at the papillary level) corresponding to our geometry: the level between the base and mid, referred here as "next to base" – node 21. The length was derived from the base to apex level. Wall thickness was computed as an average of the thickness at the base, next to base, mid and apex levels for all unloaded geometries. End diastolic volume was a direct output from Continuity based on the origin of the mesh.

For the 24 geometries, Z-scores were computed comparing LV dimensions and measures of end diastolic function for the unloaded and loaded state at mid-gestation to determine the magnitude of deviation from the measured mean. A Z-score is defined as

$$z - score = (x - \mu) / \sigma$$

where x is the observed measurement, μ is the expected measurement (experimental mean) and σ is the standard deviation of the population. Z-scores above the population mean have a positive value and those below the population mean have a negative value. While the sign indicates direction of deviation from the mean, the Z-score value conveys magnitude of deviation, which is of more interest for the statistical analyses in this study. Hence, absolute z-scores were used in calculations whereas plots were based on the raw z-score values.

With all criteria weighted equally, the LV geometries of the fetal heart with the cumulative minimum Z-score that best fit the described data for geometric and functional measures at mid-gestation were identified as the starting models for normal human fetal growth. Finally, LV short- and long-axis dimension data were extracted for each of the FE models from mid-gestation to birth. Of these, the FE model that best predicted shape for the entire gestational period, as scored using least z-scores, was identified as the working reference model for normal human fetal growth for the remainder of this study. The FE model with the lowest cumulative z-scores was chosen as the reference model for normal human fetal growth.

Supplemental Tables

Coefficient	Description	Value
Cpas [kPa]	Passive stress scaling constant	0.33
bf	Fiber strain coefficient	9.2
bc	Cross-fiber strain coefficient	2.0
bfr	Shear-strain coefficient	3.7
Ccomp	Bulk modulus	350

Supplemental Table S1: Passive Material Properties

Coefficient	Description	Value
Eff,set	Homeostatic set point for fiber strains	0.0
Ecc,set	Homeostatic set point for cross-fiber strains	0.0
beta_l	Growth rate constant in fiber direction	0.0008
beta_t	Growth rate constant in cross fiber direction	0.00026667

Supplemental Table S2: Growth Parameters

References

1. Guccione JM, McCulloch AD, Waldman LK. Passive material properties of intact ventricular myocardium determined from a cylindrical model. *J Biomech Eng* (1991) **113**:42–55.

2. Johnson P, Maxwell DJ, Tynan MJ, Allan LD. Intracardiac pressures in the human fetus. *Heart* (2000) **84**:59–63.

3. Kenny JF, Plappert T, Doubilet P, Saltzman DH, Cartier M, Zollars L, Leatherman GF, St John Sutton MG. Changes in intracardiac blood flow velocities and right and left ventricular stroke volumes with gestational age in the normal human fetus: a prospective Doppler echocardiographic study. *Circulation* (1986) **74**:1208–1216.

4. Rodriguez EK, Hoger A, McCulloch AD. Stress-dependent finite growth in soft elastic tissues. *Journal of Biomechanics* (1994) **27**:455–467.

5. Kroon W, Delhaas T, Arts T, Bovendeerd P. Computational modeling of volumetric soft tissue growth: application to the cardiac left ventricle. *Biomech Model Mechanobiol* (2009) **8**:301–309. doi:10.1007/s10237-008-0136-z

6. Göktepe S, Abilez OJ, Parker KK, Kuhl E. A multiscale model for eccentric and concentric cardiac growth through sarcomerogenesis. *Journal of Theoretical Biology* (2010) **265**:433–442. doi:10.1016/j.jtbi.2010.04.023

7. Kerckhoffs RCP, Omens J, McCulloch AD. A single strain-based growth law predicts concentric and eccentric cardiac growth during pressure and volume overload. *Mech Res Commun* (2012) **42**:40–50. doi:10.1016/j.mechrescom.2011.11.004

8. Gopalan SM, Flaim C, Bhatia SN, Hoshijima M, Knoell R, Chien KR, Omens JH, McCulloch AD. Anisotropic stretch-induced hypertrophy in neonatal ventricular myocytes micropatterned on deformable elastomers. *Biotechnol Bioeng* (2003) **81**:578–587. doi:10.1002/bit.10506

9. Russell B, Curtis MW, Koshman YE. Mechanical stress-induced sarcomere assembly for cardiac muscle growth in length and width. *Journal of molecular and ...* (2010)

10. Simpson DG, Majeski M, Borg TK, Terracio L. Regulation of cardiac myocyte protein turnover and myofibrillar structure in vitro by specific directions of stretch. *Circulation Research* (1999) **85**:e59–69.

11. Kerckhoffs RCP. Computational modeling of cardiac growth in the post-natal rat with a strain-based growth law. *Journal of Biomechanics* (2012) **45**:865–871. doi:10.1016/j.jbiomech.2011.11.028

12. McElhinney DB, Marshall AC, Wilkins-Haug LE, Brown DW, Benson CB, Silva V, Marx GR, Mizrahi-Arnaud A, Lock JE, Tworetzky W. Predictors of technical success and postnatal biventricular outcome after in utero aortic valvuloplasty for aortic stenosis with evolving hypoplastic left heart syndrome. *Circulation* (2009) **120**:1482–1490. doi:10.1161/CIRCULATIONAHA.109.848994

13. Arteaga-Martínez M, Halley-Castillo E, García-Peláez I, Villasis-Keever MA, Aguirre OM, Vizcaino-Alarcón A. Morphometric study of the ventricular segment of the human fetal heart between 13 and 20 weeks' gestation. *Fetal Pediatr Pathol* (2009) **28**:78–94. doi:10.1080/15513810802679449
